# Silver Complexes of Miconazole and Metronidazole: Potential Candidates for Melanoma Treatment

**DOI:** 10.3390/ijms25105081

**Published:** 2024-05-07

**Authors:** Małgorzata Fabijańska, Agnieszka J. Rybarczyk-Pirek, Justyna Dominikowska, Karolina Stryjska, Dominik Żyro, Magdalena Markowicz-Piasecka, Małgorzata Iwona Szynkowska-Jóźwik, Justyn Ochocki, Joanna Sikora

**Affiliations:** 1 Department of Bioinorganic Chemistry, Medical University of Lodz, Muszynskiego 1, 90-151 Lodz, Poland; karolina.stryjska@umed.lodz.pl (K.S.); dominik.zyro@umed.lodz.pl (D.Ż.); 2 Theoretical and Structural Chemistry Group, Department of Physical Chemistry, Faculty of Chemistry, University of Lodz, Pomorska 163/165, 90-236 Lodz, Poland; agnieszka.rybarczyk@chemia.uni.lodz.pl (A.J.R.-P.); justyna.dominikowska@chemia.uni.lodz.pl (J.D.); 3 Department of Applied Pharmacy, Medical University of Lodz, Muszynskiego 1, 90-151 Lodz, Poland; magdalena.markowicz@umed.lodz.pl; 4 Faculty of Chemistry, Institute of General and Ecological Chemistry, Lodz University of Technology, Zeromskiego 116, 90-543 Lodz, Poland; malgorzata.szynkowska@p.lodz.pl; 5 Faculty of Pharmacy, Chair of Medicinal Chemistry, Group of Bioinorganic Chemistry Medical University of Lodz, Muszynskiego 1, 90-151 Lodz, Poland; justyn.ochocki@umed.lodz.pl

**Keywords:** melanoma, cytotoxicity, biocompatibility, silver(I) complexes, metronidazole, miconazole, medicinal chemistry, anticancer

## Abstract

Melanoma, arguably the deadliest form of skin cancer, is responsible for the majority of skin-cancer-related fatalities. Innovative strategies concentrate on new therapies that avoid the undesirable effects of pharmacological or medical treatment. This article discusses the chemical structures of [(MTZ)_2_AgNO_3_], [(MTZ)_2_Ag]_2_SO_4_, [Ag(MCZ)_2_NO_3_], [Ag(MCZ)_2_BF_4_], [Ag(MCZ)_2_SbF_6_] and [Ag(MCZ)_2_ClO_4_] (MTZ—metronidazole; MCZ—miconazole) silver(I) compounds and the possible relationship between the molecules and their cytostatic activity against melanoma cells. Molecular Hirshfeld surface analysis and computational methods were used to examine the possible association between the structure and anticancer activity of the silver(I) complexes and compare the cytotoxicity of the silver(I) complexes of metronidazole and miconazole with that of silver(I) nitrate, cisplatin, metronidazole and miconazole complexes against A375 and BJ cells. Additionally, these preliminary biological studies found the greatest IC_50_ values against the A375 line were demonstrated by [Ag(MCZ)_2_NO_3_] and [(MTZ)_2_AgNO_3_]. The compound [(MTZ)_2_AgNO_3_] was three-fold more toxic to the A375 cells than the reference (cisplatin) and 15 times more cytotoxic against the A375 cells than the normal BJ cells. Complexes of metronidazole with Ag(I) are considered biocompatible at a concentration below 50 µmol/L.

## 1. Introduction

Skin cancers are common malignancies, with the third most common being melanoma, a condition characterized by considerable heterogeneity and a propensity to metastasize to distant organs [1]. However, its precise development remains unknown. Its genesis has been attributed to the harmful effects of ultraviolet rays and genetic factors. Melanoma has a high metastatic potential, resulting in its very limited response to current therapies, which for many years were limited to surgery, radiotherapy and chemotherapy. However, recent advances in knowledge about the pathophysiological mechanisms of the disease have allowed for the development of new therapeutic classes, such as immune checkpoint and small-molecule kinase inhibitors [2,3].

Melanoma has low sensitivity to known cytostatic drugs. These cancer cells demonstrate a significant ability to self-repair DNA damage and express high levels of proteins involved in the elimination of xenobiotics. The initiation and progression of melanoma are mediated via genetic and epigenetic alterations to the key molecules in multiple signaling pathways, such as the RAS/RAF/MAPK, JNK, PI3K/Akt and Jak/STAT pathways; they are also influenced by the dysregulation of the MITF (Microphthalmia-Associated Transcription Factor) protein [4,5,6,7]. This property of melanoma cells strongly inhibits the effect of chemotherapeutics. Nevertheless, the arsenal of biological drugs for the treatment of melanoma is extensive and continues to grow, and great progress has been observed in the number of immunotherapy agents available for melanoma treatment, with substantially improved outcomes [8]. Even so, despite these successes, many patients will not benefit from immunotherapy or will experience tumor growth after an initial response. In such cases, traditional chemotherapy, as monotherapy or as combination therapy, is used as an addition to treatment. Novel therapeutic strategies are still in progresses for melanoma, and drug combinations appear to represent the most logical and rational approach to helping patients overcome ineffective treatments or treatment resistance, ultimately improving their survival and quality of life.

An alternative lies in the use of silver(I) complexes, which can have both antibacterial and cytotoxic properties. A considerable number of studies indicate that transition metal ions may have a role in medicine and have demonstrated their innovative therapeutic effects, presenting them as an alternative in combating infections [9]. In particular, silver(I) ions and their complexes exhibit antimicrobial, antioxidant and cytotoxic effects, which have been well established in medicine for centuries [10]. Silver has a lower toxicity than other transition metals and has been utilized to treat skin ulcers and burns, as well as for gastric treatment and to sterilize medical instruments [11,12,13]. It has been proven that silver(I) complexes have superior biological effects, including genotoxicity, cytotoxicity and antimicrobial activity, compared to the free ligand [14,15,16,17].

**Figure 1:**
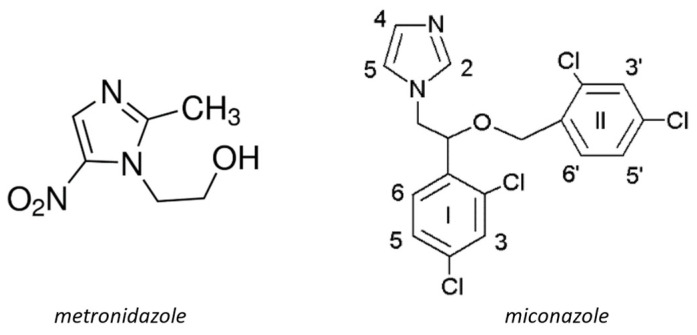
Chemical structure of the ligands: metronidazole (MTZ) and miconazole (MCZ).

Moreover, silver(I) complexes with metronidazole (MTZ) and miconazole (MCZ) have demonstrated more diverse biochemical properties compared to free ligands and silver(I) salts alone [18,19,20,21,22,23]. Miconazole (Figure 1) is a broad-spectrum azole antifungal with some additional activity against Gram-positive bacteria. It is widely used to treat mucosal yeast infections, including both oral and vaginal infections; although intravenous miconazole is no longer available, a wide variety of suppositories, creams, gels and tablet-based products are still in use [24]. Studies investigating the antitumor properties of miconazole were reported in 1991 [25]. Miconazole is a potent inhibitor of CYP2C, which is involved in cancer formation and progression. CYP2C9 oxidizes arachidonic acid (ARA) into epoxyeicosatrienoic acids (EETs) and promotes mitogenesis and angiogenesis. The inhibition of CYP2C9 suppresses EET biosynthesis, subsequently reducing the proliferation and migration of human endothelial cells [26,27]. Recent studies have indicated that miconazole also exhibits potent anticancer effects in various types of cancer via apoptosis activation; more precisely, miconazole induced autophagy-mediated cell death in glioblastoma cell lines with intracellular ROS production and ER stress [28]. Studies on apoptosis induction through the death receptor 5-dependent and mitochondrial-mediated pathways in human bladder cancer cells found that MCZ inhibited cancer cell growth in COLO 205 cells, with the cells arrested at the G0/G1 phase of the cell cycle [29]. In addition, MCZ treatment yielded significant therapeutic effects in vivo in nude mice bearing COLO 205 tumor xenografts [30].

Metronidazole (Figure 1) is a commonly used nitroimidazole antibiotic. It is frequently used to treat gastrointestinal infections and parasitic infections such as trichomoniasis, giardiasis and amebiasis [31,32]. Metronidazole is considered to have anti-tumor activity due to its ability to penetrate hypoxic tumors and accumulate [33]. Nitroimidazoles can act as oxygen mimics, especially in hypoxic cells, so they can be used during irradiation to fix radiation-induced damage in DNA or other macromolecules [34]. It has also been reported that some derivatives of 2-nitroimidazole inhibited tumor-specific angiogenesis by blocking the production of angiogenic factors [35]. However, metronidazole possesses less cytotoxicity than doxorubicin, and its nanocomposites demonstrated very little toxic action toward HEK 293 cells. This is an important consideration when applying metronidazole composites with silver nanoparticles as antibacterials, as they do not demonstrate toxic side effects toward mammalian cells and tissues [36].

The present article discusses the chemical structure of ten silver(I) complexes with miconazole and metronidazole. The six silver(I) compounds, viz. [(MTZ)_2_AgNO_3_], [(MTZ)_2_Ag]_2_SO_4_, [Ag(MCZ)_2_NO_3_], [Ag(MCZ)_2_BF_4_], [Ag(MCZ)_2_SbF_6_] and [Ag(MCZ)_2_ClO_4_], were synthetized by our group and further studied [18,19,20,21,22,23], along with their potential cytostatic activity in A375 human skin cancer cells and normal BJ cells. In addition, to comprehensively assess their potential as anticancer agents, this study also evaluates their impact on blood coagulation and red blood cell hemolysis. As most anticancer drugs are introduced via intravenous injection, resulting in instantaneous contact with blood, the study assumes that their interaction with various biological systems, including the coagulation system, blood platelets and plasma, is also instantaneous.

## 2. Results

### 2.1. Structural Analysis

**Figure 2:**
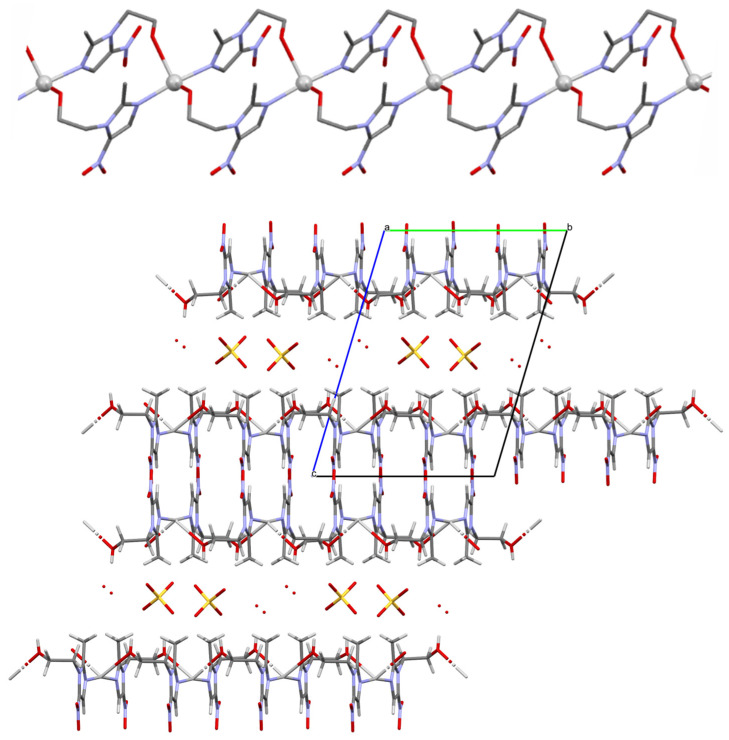
Polymeric structure of the [Ag(MTZ)_2_]_2_SO_4_ compound. The structure of a single [Ag(MTZ)_2_]^+^ chain (at the top) and intertwining cationic chains, anions and water molecules in the crystal structure within a unit cell. Silver atoms, sulfate anions and the oxygen atoms of water are presented in ball-and-stick style. Elements are represented using the following colors: Ag—light gray, C—dark gray O—red, N—blue, S—yellow. H atoms are omitted for clarity.

The group of studied compounds includes four miconazole and six metronidazole silver(I) complexes. Their crystals represent typical salt structures. Miconazole and metronidazole ligands are integrated into the coordination of silver cations via the nitrogen atoms of imidazole rings. The longest bonds with the metal cation (of the Ag-N type) are observed in miconazole nitrate and metronidazole complexes (trifluoromethylcarboxylate, nitrate, sulfate and methylsulfate). Almost all the compounds also exhibit close contact between the silver atom and the surrounding atoms of the anionic ligands of the neighboring coordination sphere (Table 1). In all the mentioned cases, these distances are below 3Å and disturb slightly the linear structure of the complex, resulting in N-Ag-N angle values varying from 152° to 180°. The structure of metronidazole silver(I) sulfate [Ag(MTZ)_2_]_2_SO_4_ differs significantly from the other complex salts because its anions are not arranged in the coordination sphere of the silver atoms. Instead, the hydroxyl and nitro groups of the neighboring metronidazole ligands contribute to the coordinating silver ion, thus forming a polymeric, positively charged structure. Metronidazole ligands with silver cations [Ag(MTZ)_2_]^+^ form infinite ladder-like cationic chains; these are arranged in parallel layers, intertwined with counter ions and water molecules (Figure 2).

**Figure 3:**
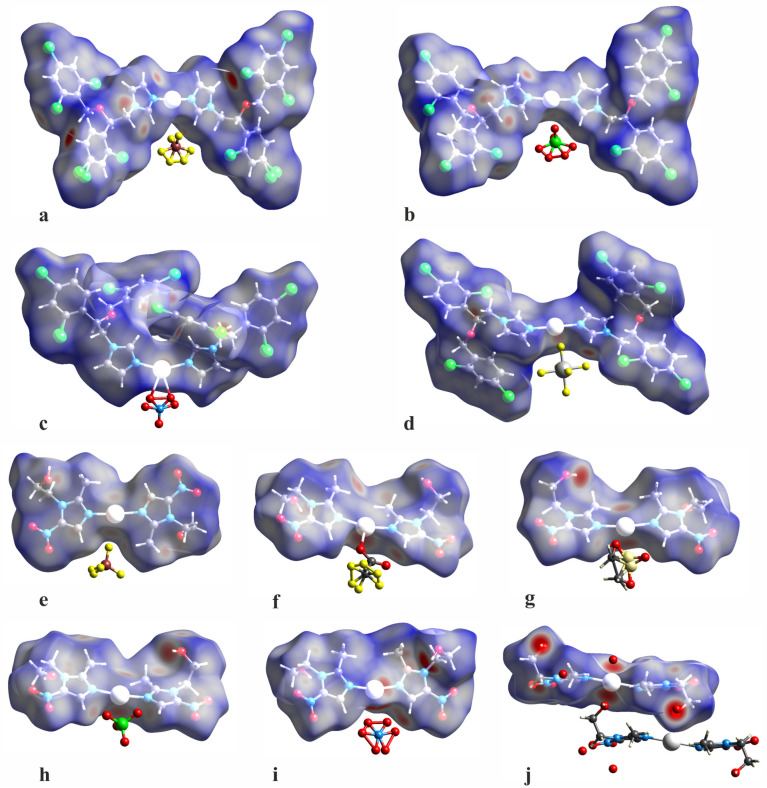
Molecular Hirshfeld surfaces of the studied complexes in the crystalline state: [Ag(MCZ)_2_BF_4_] (**a**), [Ag(MCZ)_2_ClO_4_] (**b**), [Ag(MCZ)_2_NO_3_] (**c**), [Ag(MCZ)_2_SbF_6_] (**d**), [Ag(MTZ)_2_BF_4_] (**e**), [Ag(MTZ)_2_CF_3_CO_2_] (**f**), [Ag(MTZ)_2_CH_3_SO_3_] (**g**), [Ag(MTZ)_2_ClO_4_] (**h**), [Ag(MTZ)_2_NO_3_] (**i**), [Ag(MTZ)_2_]_2_SO_4_ (**j**); appropriate colors indicate the following elements: dark gray—C, white—H, blue—N, light gray—Ag, red—O, brown—B, green—C, yellow—F, dark gray—Sb; green—Cl. For the complex ions, the Hirshfeld surfaces are mapped with the d_norm_ parameter using a color scale: red color indicates intermolecular contact areas shorter than the sum of the van der Waals radii.

The intermolecular contacts in the crystal structures can be identified in more detail using Hirshfeld surface analysis. Figure 3 presents the molecular Hirshfeld surfaces calculated for the complex cations ([Ag(MTZ)_2_]^+^ and [Ag(MTZ)_2_]^+^) in all the investigated crystal structures. They are mapped according to the color scale of the normalized contact distance d_norm_. Red indicates areas on the surface which correspond to very short intermolecular contacts and therefore are important in crystal packing and intermolecular interactions. As mentioned, the sulphate differs in structure from the others: complex cations are linked to an endless polymeric structure through the hydroxyl groups of the metronidazole ligand. The effect is shown on the Hirshfeld molecular surface (k) as a series of intensive red marked areas surrounding the hydroxyl group. This type of bond to the metal ion significantly affects the character of the silver coordination sphere; however, unlike other compounds, no anions are involved.

**Figure 4:**
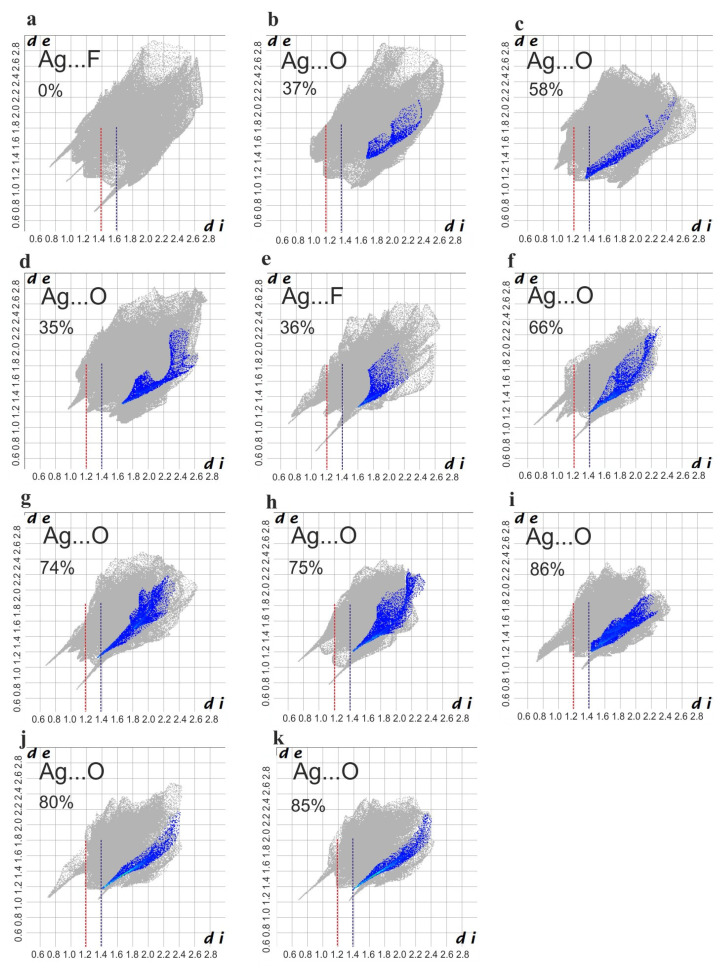
Hirshfeld fingerprint plots of the studied complexes: [Ag(MCZ)_2_BF_4_] (**a**), [Ag(MCZ)_2_ClO_4_] (**b**), [Ag(MCZ)_2_NO_3_ (**c**), [Ag(MCZ)_2_SbF_6_ (**d**), [Ag(MTZ)_2_BF_4_ (**e**), [Ag(MTZ)_2_CF_3_CO_2_ (**f**), [Ag(MTZ)_2_CH_3_SO_3_] (**g**), [Ag(MTZ)_2_ClO_4_] (**h**), [Ag(MTZ)_2_NO_3_] (**i**), [Ag(MTZ)_2_]_2_SO_4_ (**j**,**k**)—crystal contains two silver atoms in the symmetric unit, hence there are presented two fingerprint plots for two crystallographically independent silver complex molecules; only contacts of the second coordination sphere with the silver atoms are presented; the dotted line represents the contact distance between the molecular Hirshfeld surface of the silver complex cation and atoms of surrounding anions (oxygen or fluorine): red—1.2; dark blue—1.4.

Unlike miconazole sulphate, other silver complexes form finite molecular systems with a “butterfly-like” shape, which is especially clear for the miconazole complexes. This molecular shape mainly results from crystallographic two-fold symmetry. Several light red areas on the molecular surfaces are connected with hydrogen or hydrophobic short intermolecular contacts. Hirshfeld surface analysis can be also used for quantitative analysis of the intermolecular contacts. Hence, Hirshfeld surface fingerprint plots were prepared to present the relative numbers of specific intermolecular contacts (Figure 4). The contacts between the complex cations and the surrounding anions were subject to detailed inspection.

In the miconazole complexes, the second coordination sphere (Ag…O distances) generally demonstrates a relatively longer radius compared to that in the metronidazole complexes. The shortest distances are observed for both nitrate complex salts (c and i) and for the metronidazole complexes with strongly electronegative ionic ligands—[Ag(MTZ)_2_CF_3_CO_2_] (f), [Ag(MTZ)_2_CH_3_SO_3_] (g), [Ag(MTZ)_2_ClO_4_] (h), [Ag(MTZ)_2_NO_3_] (i), [Ag(MTZ)_2_]_2_SO_4_ (j). It is worth mentioning that the observed short distances are assisted by the largest percentage of contact (up to 86%) with the molecular Hirshfeld surface around the silver atom, i.e., the oxygen atoms of the anions Ag…O. As mentioned previously, such short distances are accompanied by the elongation of the Ag-N bonds of the first coordination sphere.

To clarify whether the changes in the structure around the silver atom are only the result of packing effects in the crystal, additional studies based on quantum chemistry were carried out for the isolated complex cation–anion systems in the gaseous state. The last compound (sulphate), for which the X-ray analyses did not determine the structure of the simple complex ion–anion system, was excluded from the study.

### 2.2. Computational Results

In some cases, the atoms of anions (e.g., oxygen or fluorine) exhibit positional disorders in accordance with the symmetry of their crystals. Quantum–chemical calculations were therefore undertaken to provide structural insight into atoms with specific atomic positions. In all cases, the major disorder component was taken into account.

The position of the silver atom plays a key role in determining the cytotoxicity of a complex, being responsible for the possible detachment of the ligands in solution. Its selected geometrical parameters in the optimized structures are listed in Table 2.

The Ag-N bond lengths in the studied structures range between 2.107 Å (for [Ag(MCZ)_2_SbF_6_]) and 2.205 Å (for [Ag(MTZ)_2_CF_3_CO_2_]), and the values of the N-Ag-N angle are in the range of 157.3° (for [Ag(MTZ)_2_CF_3_CO_2_]) to 179.1° (for [Ag(MTZ)_2_ClO_4_]). No straightforward relationship was observed between either the Ag-N distance or the N-Ag-N angle and the type of ligand (MCZ or MTZ) binding the silver(I) cation. However, in most cases, shorter Ag-N distances correlated with larger N-Ag-N angles (the spatial arrangement is closer to linear), signifying an increase in the role of dsp^3^ hybridization for the Ag cation. This observation is in agreement with the X-ray experimental results.

**Figure 5:**
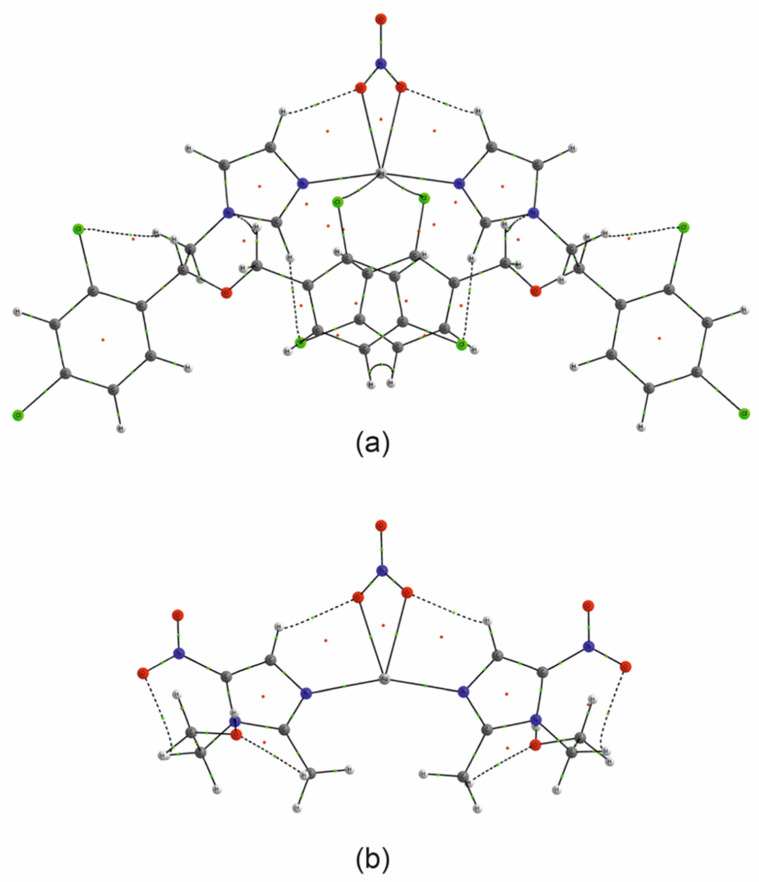
Molecular graphs of [Ag(MCZ)_2_NO_3_] (**a**) and [Ag(MTZ)_2_NO_3_] (**b**); green dots—bond critical points, red dots—ring critical points; cage critical points are omitted for clarity.

The results of the structural and electron density analyses may complement each other. The values of electron density (ρ), its Laplacian ($\nabla^{2}\rho$) and the total energy density (H) at the Ag-N bond critical points (BCPs), calculated according to the framework of QTAIM theory, are given in Table 3. Molecular graphs of the complexes are shown in Figure 5 for the nitrates and in Figures S1–S7 in the Supplementary Materials for the rest of the studied complexes.

One may easily notice that for systems with longer Ag-N contacts and smaller N-Ag-N angles, the electron density values and their Laplacians are lower than those of shorter Ag-N contacts with a more linear arrangement. The absolute total energy density values (negative for all systems) follow the same trend.

The highest occupied molecular orbital (HOMO) and lowest unoccupied molecular orbital (LUMO) energies were measured, as the size of the energy gap between them (HOMO-LUMO) is known to be connected with biological activity [37]. The HOMO (E_HOMO_) and LUMO (E_LUMO_) energies and the HOMO-LUMO (ΔE_HOMO/LUMO_) gap values are listed in Table 4**.**

Interestingly, the smallest HOMO-LUMO gap values can be seen for the systems with longer Ag-N contacts and smaller N-Ag-N angles and electron density parameters, viz. density, its Laplacian value and absolute total energy density. Generally, one may say that systems exhibiting a smaller HOMO-LUMO energy gap show greater biological activity. This feature has been demonstrated for pyrrole-2,5-dione analogs [38].

Later in this study (paragraph 2.3), the IC_50_ data will be presented. The HOMO-LUMO energy gap values (Table 4) indicate that [Ag(MCZ)_2_NO_3_**]**, [Ag(MTZ)_2_NO_3_**],** [Ag(MTZ)_2_CH_3_SO_3_**]** and [Ag(MTZ)_2_CF_3_CO_2_**]** may exhibit the desired cytotoxic activity because they show smaller HOMO-LUMO energy gaps. However, such predictions should be made with care, as a huge variety of factors are known to affect cytotoxicity in biological systems.

Summarizing the results of the electron density analysis of the miconazole complexes, it was found that for both nitrates, the Ag–N bonds differed from those of the remaining complex ions. A similar situation was observed for the following metronidazole complexes: [Ag(MTZ)_2_CH_3_SO_3_], [Ag(MTZ)_2_CF_3_CO_2_] and [Ag(MTZ)_2_NO_3_]. The electron density at the critical point of the bond is more than 0.0100 e Å^−3^ lower than that of the other systems. Also, the total energy density (negative) at the bond critical point is closer to zero for the nitrates. These results indicate that the metal–ligand bonds in these systems are characterized by a lower energy, they may be easier to break and the silver ion could be released more easily from the complex. It is therefore possible that the obtained results are consistent with those of our experimental studies, further described in detail in Section 2.3, i.e., in terms of the length of the Ag–N bonds in the crystalline state, as well as the observed activity of the nitrate compounds: the other metronidazole complexes were not tested against the cancer cells.

### 2.3. Cytotoxicity Evaluation

The cytotoxicity of the compounds MTZ, MCZ, [(MTZ)_2_AgNO_3_], [(MTZ)_2_Ag]_2_SO_4_, [Ag(MCZ)_2_NO_3_], [Ag(MCZ)_2_BF_4_], [Ag(MCZ)_2_SbF_6_] and [Ag(MCZ)_2_ClO_4_] was then tested against skin cancer and normal cells, with the anticancer drug cisplatin as a reference. The cytotoxic activity was measured against the melanoma A375 tumor cell line (ECACC) and the human BJ fibroblast cell line (ATCC) using an MTT reduction assay. The results are given in Table 5. The selectivity index (SI) was calculated using the following formula: SI = IC_50_ (BJ)/ IC_50_ (A375). SI values higher than 1 indicate that a compound exhibits greater activity against cancer cells than healthy cells, which is desirable. Silver(I) nitrate and its complexes evoked a dose-dependent reduction in A375 cell viability after 72 h of exposure, as did miconazole. Cisplatin exhibited a dose-dependent cytotoxic effect on both of the tested cell lines. The greatest decline in A375 cell viability was observed for [Ag(MCZ)_2_NO_3_] and [(MTZ)_2_AgNO_3_] (IC_50_ values of 1.7 μM and 3 μM, respectively). Against the A375 cells, the silver(I) complex [(MTZ)_2_AgNO_3_] was three-fold more toxic than cisplatin and seven-fold more toxic than [(MTZ)_2_Ag]_2_SO_4_. Also, the [(MTZ)_2_AgNO_3_] compound was 15 times more toxic against the cancer cell line than the normal BJ line (IC_50_ = 46 μM; SI = 15.3), which is a particularly desirable feature. This compound was also two-fold less toxic than the reference cisplatin against the normal cells tested. Previous studies have found the compound [(MTZ)_2_AgNO_3_] demonstrated higher activity towards cancer cell lines than against the normal BJ line and exhibited a six-fold higher cytotoxicity to the HepG2 liver cancer line and a three-fold higher cytotoxicity to the PANC-1 pancreatic cancer cell line than to the BJ line [18,19]. In turn, [Ag(MCZ)_2_NO_3_] demonstrated around an eight-fold higher cytotoxic activity against the melanoma lines than against the fibroblast cells (SI = 7.8) and a five-fold superiority over cisplatin. The compound [Ag(MCZ)_2_NO_3_] was around 30 times less toxic to human fibroblasts than to the HepG2 liver cancer line [22]. The remaining silver ion coordination compounds with miconazole had a similar cytotoxicity profile against both the melanoma and fibroblast lines. At this point, one should notice that the cytotoxicity results for both [(MTZ)_2_AgNO_3_] and [Ag(MCZ)_2_NO_3_] are in line with the preliminary predictions made on the basis of our previous computational studies (Section 2.2).

### 2.4. Preliminary Biocompatibility Study

The examined compounds and their ligands were evaluated for their impact on the extrinsic coagulation pathway (prothrombin time (PT)) and the intrinsic coagulation pathway (activated partial thromboplastin time (APTT)), as well as the common coagulation pathway, which involves the polymerization of fibrin (thrombin time (TT)). Their effect on human red blood cell morphology was also recorded [39]. Their influence on the overall potential for clot formation and fibrinolysis was evaluated using the CL-test. In addition, the metronidazole complexes and their ligands were evaluated in terms of their biocompatibility with model cells (erythrocytes) according to erythrotoxicity studies. It appears that solvents used for the preparation of compound solutions do not disrupt the examined biochemical processes when applied as aqueous, methanol or DMSO solutions (the DMSO content in the biological sample must be <1%). As the miconazole complexes were insoluble in water and methanol and exhibited poor solubility in dimethyl sulfoxide (DMSO), they were not tested.

**Figure 6:**
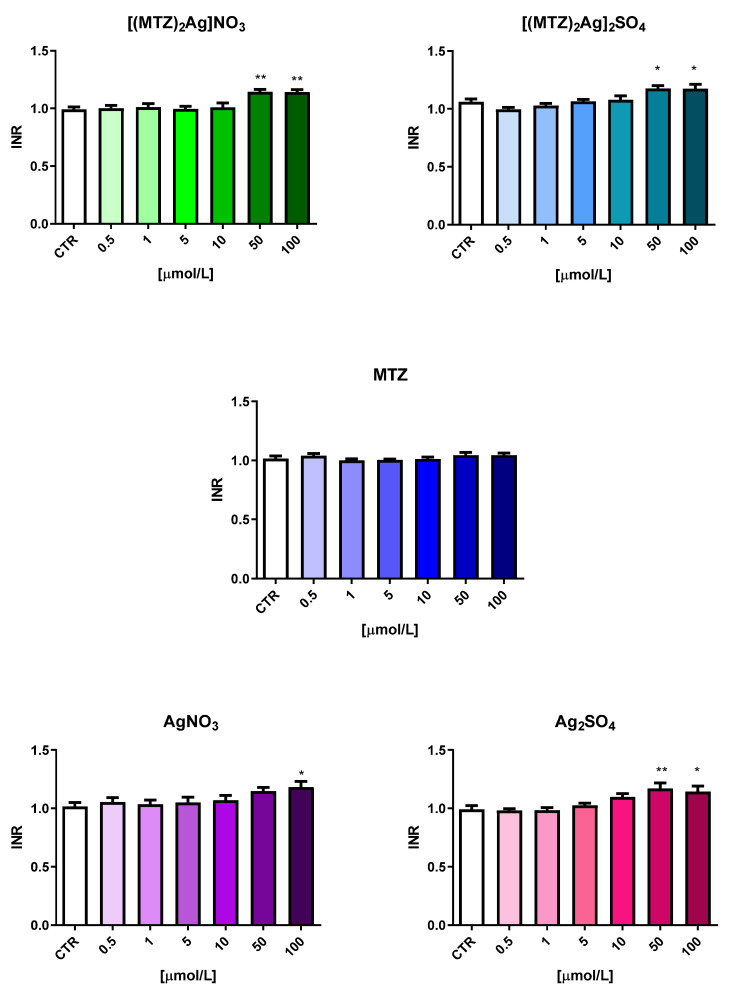
The influence of the tested silver(I) metronidazole complexes ([(MTZ)_2_AgNO_3_] and [(MTZ)_2_Ag]_2_SO_4_) and ligands (MTZ—metronidazole; AgNO_3_; Ag_2_SO_4_) on prothrombin time (PT) (reference range: 9.7–14.6 s). The results are presented as mean ± standard deviation (*n* = 5); * *p* < 0.05, ** *p* < 0.01 indicates a significant difference from the control sample (CTR).

**Figure 7:**
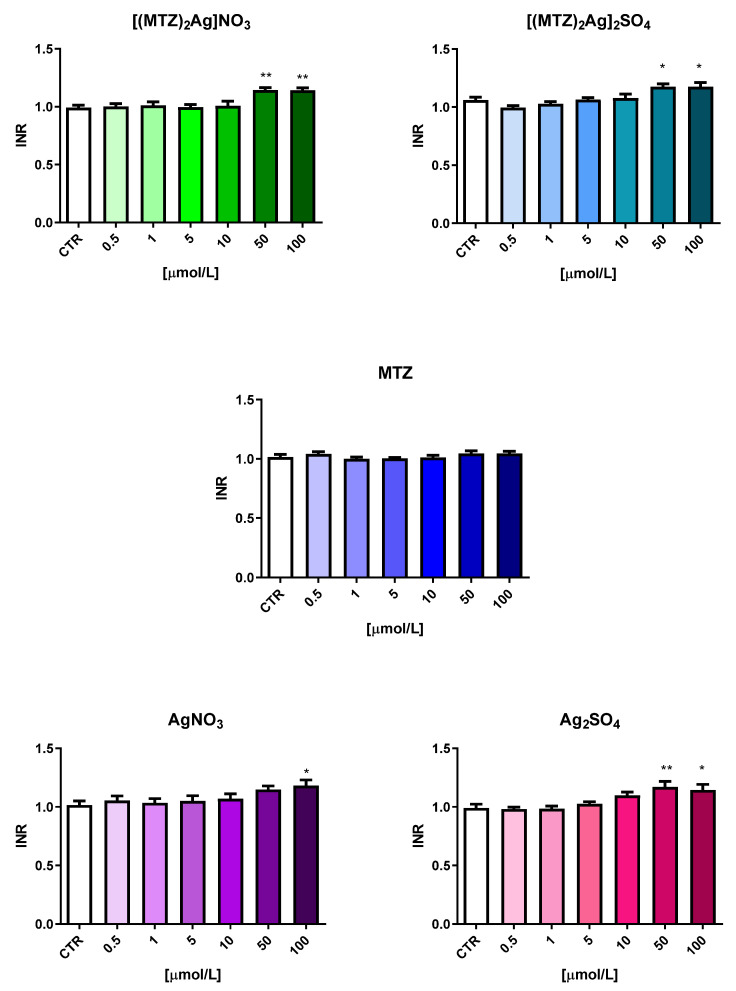
The influence of the tested silver(I) metronidazole complexes ([(MTZ)_2_AgNO_3_] and [(MTZ)_2_Ag]_2_SO_4_) and ligands (MTZ—metronidazole; AgNO_3_; Ag_2_SO_4_) on international normalized ratio (INR) (reference range: 0.8–1.2). The results are presented as means ± standard deviation (*n* = 5); * *p* < 0.05, ** *p* < 0.01 indicates a significant difference from the control sample (CTR).

The first stage assessed the changes in plasma hemostasis occurring under the influence of the five test compounds. Plasma was incubated with the tested compounds and ligands at six different concentrations ranging from 0.5 to 100 µmol/L. The plasma was obtained from five different volunteers. To evaluate the impact on the extrinsic coagulation pathway, the prothrombin time (PT) was determined in human citrated plasma, expressed in seconds and as the international normalized ratio (INR) (Figure 6 and Figure 7). Both metronidazole compounds significantly lengthened the PT as complexes at concentrations of 50.0 µmol/L and 100 µmol/L and when using Ag_2_SO_4_ at a concentration of 100 µmol/L. However, despite being statistically significant, the observed changes did not exceed the range of reference values for either the mean PT (reference range: 9.7–14.6 s) or INR (reference range: 0.8–1.2). Such results may indicate that even though high concentrations of these compounds can influence the factor VIIa-dependent coagulation process, they do not interfere with the intrinsic pathway: even at such high concentrations, they do not cause a pathological prolongation of the PT or INR, i.e., beyond the reference range.

To evaluate the intrinsic coagulation pathway, the plasma was mixed with the tested compounds at the same concentrations as in the PT analysis, and the activated partial thromboplastin time (APTT) was measured. Only the metronidazole complex with Ag_2_SO_4_ at the highest concentration (100 µmol/L) significantly prolonged the APTT; however, the mean APTT did not exceed the reference range (26.7–40.0 s) (Figure S8).

The common pathway was assessed based on thrombin time (TT). None of the tested compounds showed a statistically significant impact on the TT (Figure S9). Thrombin time can be used to assess the rate of the conversion of fibrinogen into fibrin under the influence of exogenous thrombin and Ca^2+^ ions. In vitro studies suggest that changes in this parameter could indicate an interaction between the tested compound and fibrinogen or a polymerization process involving the binding of fibrin fibers. Following this, the impact of the tested complex compounds and their ligands on the overall clot formation and fibrinolysis potential (CL_AUC_) and individual kinetic parameters was assessed using the CL-test. The analysis was performed using plasma from three different donors. The kinetic parameters associated with clot formation and lysis for both the examined complex compounds are presented in Table 6 and Table 7. No statistically significant changes were observed in either CL_AUC_ or the other parameters, indicating that neither compound disrupted the clot formation process nor its stabilization phase or lysis. Interestingly, in all the examined cases, complete lysis of the clots was observed, indicating that the Fmax and Lmax values for each curve did not differ significantly. Similarly, no significant differences were observed for MTZ or AgNO_3_ in the Ag_2_SO_4_ complexes (data not published, available from the authors).

Their erythrotoxicity was also assessed. Red blood cells are model cells used in basic studies of the biocompatibility of new compounds and some medical materials. Through these analyses, we can preliminarily assess whether a new compound damages the cell membrane and in what concentration range erythrotoxic effects are not observed. A compound is considered biocompatible when it does not cause hemolysis greater than 10% [40]. The amount of hemoglobin released from damaged cells was measured using a spectrophotometer.

**Figure 8:**
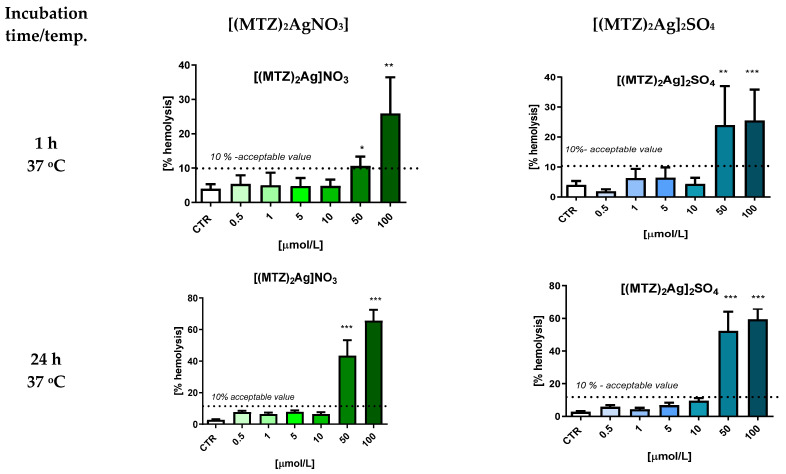
The effects of incubation of 2% RBC suspension with the tested silver(I) metronidazole complexes ([(MTZ)_2_AgNO_3_] and [(MTZ)_2_Ag]_2_SO_4_) on RBC hemolysis (acceptable value of hemolysis proving the biocompatibility of the compound is 10%). The results are presented as means ± standard deviation (*n* = 3); * *p* < 0.05, ** *p* < 0.01, *** *p* < 0.01 indicates a significant difference from the control sample (CTR).

Erythrotoxicity was assessed in a 2% erythrocyte suspension following 1 h and 24 h of incubation with the tested compounds. After one hour, significant changes in erythrocyte hemolysis (26% to 31%) were observed for the highest concentrations of [(MTZ)_2_AgNO_3_] and [(MTZ)_2_Ag]_2_SO_4_] (Figure 8) and for the ligands AgNO_3_ and Ag_2_SO_4_ at 100 µmol/L (Figure S10). After 24 h, a significantly increased degree of hemolysis (>60%) was observed for the AgNO_3_ and Ag_2_SO_4_ complexes and AgNO_3_ and Ag_2_SO_4_ alone at concentrations of 50 µmol/L and 100 µmol/L). The metronidazole complexes and inorganic ligands demonstrated biocompatibility at concentrations below 50 µmol/L. However, metronidazole had no effect on the integrity of the erythrocyte protein–lipid membrane at any concentration at either time point.

**Figure 9:**
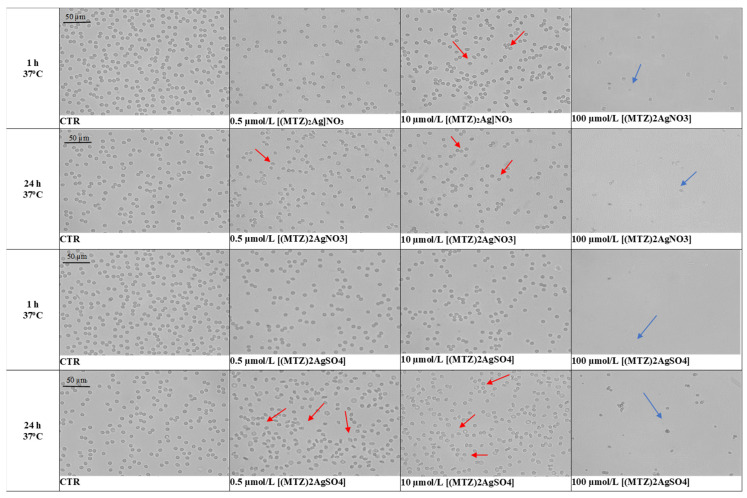
Effects of the tested silver(I) metronidazole complexes ([(MTZ)_2_AgNO_3_] and [(MTZ)_2_Ag]_2_SO_4_]) on the morphology of red blood cells in vitro after one hour and 24 h of incubation at 37 °C. Representative phase contrast images are shown (magnification = 400 times); red arrows indicate echinocytes, blue arrows cell fragments.

Microscopic assessment of the erythrocyte morphology indicated the presence of echinocytes following 1 h and 24 h of incubation (Figure 9). Their number was found to increase with the concentration and incubation time. The presence of echinocytes confirms that the compounds interacted with the erythrocyte membrane, resulting in a more folded erythrocyte membrane and a greater number of protrusions. In the tests at a concentration of 100 nmol/L, significantly reduced numbers of red blood cells in the visual field and visible membrane fragments were observed, indicating the breakdown of the erythrocyte membrane. After 24 h, echinocytes were observed for both tested compounds. In some of the tested samples, a significantly reduced number of red blood cells was observed, which also correlated with the increased degree of hemolysis observed in the erythrotoxicity test. The influence of the ligands on red blood cells was also assessed (unpublished data, available from the authors).

To summarize, after 24 h of incubation, all the tested compounds penetrated the cell membrane, resulting in the appearance of echinocytes, and the complexes of metronidazole with Ag(I) are considered biocompatible (hemolysis below 10%) at concentrations below 50 µmol/L.

## 3. Materials and Methods

### 3.1. Cambridge Structural Database Search

The Cambridge Structural Database [41] was searched for structural data on the following silver miconazole (MCZ) and metronidazole (MTZ) ligand compounds. Ten complexes were found: four with miconazole, viz. [Ag(MCZ)_2_BF_4_], [Ag(MCZ)_2_ClO_4_], [Ag(MCZ)_2_NO_3_] and [Ag(MCZ)_2_SbF_6_], and six with metronidazole, viz. [Ag(MTZ)_2_BF_4_], [Ag(MTZ)_2_CF_3_CO_2_], [Ag(MTZ)_2_CH_3_SO_3_], [Ag(MTZ)_2_ClO_4_], [Ag(MTZ)_2_NO_3_] and [Ag(MTZ)_2_]_2_SO_4_. These were subjected to comparative structural analysis. In several cases, the anions display positional disorder resulting from the crystallographic symmetry of the complex.

### 3.2. Hirshfeld Surface Analysis

The molecular Hirshfeld surfaces of the [Ag(MTZ)_2_]^+^ and [Ag(MTZ)_2_]^+^ cations were generated using the automatic procedures implemented using CrystalExplorer 3.0 [42]. The surfaces were mapped with d_norm_, a parameter which reflects intermolecular distances; the results are presented as colors ranging from −1.0 (red), indicating distances shorter than the sum of the van der Waals radii, to 2.0 (blue)m indicating distances longer than the sum of the van der Waals radii. The Hirshfeld surface fingerprint plots include the intermolecular contacts between the silver and oxygen/fluorine atoms of the complexation anions. The presented percentage vales represent the ratio of the contact under investigation to all the contacts of the silver atoms [43].

### 3.3. Computational Methods

The structures of the ten complexes containing a single silver atom, viz. [Ag(MCZ)_2_BF_4_], [Ag(MCZ)_2_ClO_4_], [Ag(MCZ)_2_NO_3_], [Ag(MCZ)_2_SbF_6_], [Ag(MTZ)_2_BF_4_], [Ag(MTZ)_2_CF_3_CO_2_], [Ag(MTZ)_2_CH_3_SO_3_], [Ag(MTZ)_2_ClO_4_], [Ag(MTZ)_2_NO_3_] and [(MTZ)_2_Ag]_2_SO_4_, were optimized at the ωB97X-D/def2-TZVPP level [44,45,46]. The def2-TZVPP basis set includes pseudopotentials accounting for relativistic effects on the silver atoms and antimony. The stationary points were subjected to frequency analysis at the same level of theory to verify whether the optimized geometries correspond to potential energy surface minima; no imaginary frequencies were found. The above calculations were performed with Gaussian 16 software [47]. For the optimized structures, selected structural parameters were analyzed, as well as the energy difference between the lowest unoccupied (LUMO) and highest occupied molecular orbital (HOMO), called the HOMO-LUMO gap. An analysis of the electron density distribution based on QTAIM theory [48] was also carried out for the optimized geometries of the silver(I) complex salts. The QTAIM analysis generated a wealth of information concerning the intra- and intermolecular interactions, such as the electron density (ρ) or the Laplacian of the electron density (∇^2^ρ) calculated at specific points of the molecular space; the latter parameter describes the local charge concentration and depletion. The QTAIM method can also be used to characterize and define various types of closed- and shared-shell bonding [49,50,51]. In the present study, QTAIM calculations were performed to determine the nature of the interatomic bonding around the silver center in the investigated systems using AIMAll software [52].

### 3.4. Cytotoxicity Evaluation

The A375 cell line was purchased from the European Collection of Authenticated Cell Cultures (ECACC, Salisbury, UK) and the BJ human fibroblast cell line from the American Type Culture Collection (ATCC, Manassas, VA, USA). All the cell lines were grown as a monolayer in standard conditions: 37 °C, 100% humidity, under an atmosphere of 5% CO_2_. The A375 cells were cultured in Dulbecco’s Modified Eagle Medium supplemented with 15% fetal calf serum and 50 IU/mL penicillin/streptomycin. The BJ cell line was grown in EMEM with EBSS, NEAA and sodium pyruvate with L-Glutamine supplemented, 10% fetal bovine serum and 50 IU/mL penicillin/streptomycin.

The cytotoxicity of the silver(I) complexes and silver nitrate (AgNO_3_), metronidazole and miconazole was determined against the BJ and A375 cells using an MTT (3-(4,5 dimethyl-thiazol-2-yl)-2, 5-diphenyl tetrazolium bromide) assay. Next, the cells were seeded in 96-well plates at a density of 5000 cells per well and incubated overnight to reach the logarithmic growth phase. Next, the studied compounds were added (0.1, 0.5, 1, 2.5, 5, 10, 25, 50 and 100 µM) and cultured for 72 h; the cell lines were also treated with cisplatin for 72 h as a positive control. After 72 h, 10 µL of MTT solution (5 g/L) was added into each well and incubated for an additional four hours. Subsequently, the medium was removed, and 100 µL of dimethyl sulfoxide (DMSO) was added into each well. The absorbance was then measured at 570 nm using a SPECTROstar Nano microplate reader (BMG Biotech Inc., Cary, NC, USA)/Tecan Spark 10M. The mean value from three independent BJ and A375 cell cultures was taken to calculate the cell viability, expressed as a percentage of the control values, i.e., untreated cells assumed as 100%.

### 3.5. Biocompatibility Studies

Biocompatibility was evaluated using human blood. The study examined the PT, APTT and TT, as well as the clot formation and fibrinolysis test (CL-Test) and interactions with the erythrocyte membrane.

#### 3.5.1. Biological Materials

The blood samples were obtained from healthy donors at the Blood Donation Centre, Lodz. All the experiments involving biological materials were approved by the Bioethics Committee of the Medical University of Lodz (Approval No. RNN/105/20/KE) and conducted in accordance with the Polish national directives. The blood samples used were residual materials from routine diagnostic studies, destined for disposal as medical waste. The blood was collected into vacuum tubes filled with 3.2% buffered sodium citrate (1:9). Platelet-poor plasma (PPP) and red blood cells were obtained using centrifugation at 3000× *g* for 10 min at room temperature using a Mikro 22R centrifuge (Hettich Zentrifugen, Tuttlingen, Germany). The plasma was used for the hemostasis tests. Before use, the centrifuged erythrocyte mass was washed three times with physiological saline solution to remove the plasma protein residues and other blood cells.

#### 3.5.2. Basic Coagulation Tests: PT, APTT and TT

The PT, APTT and TT were measured using a Coag Chrom 3003 coagulometer (Bio-Ksel, Grudziadz, Poland) according to the manufacturer’s instructions, using commercially available reagents (Bio-Ksel, Grudziadz, Poland). The experiment was conducted as three independent experiments (in duplicate); distilled water or methanol was used as the control. The results are presented as means ± standard deviation (SD).

#### 3.5.3. Clot Formation and Fibrinolysis Test (CL-Test)

The influence of the compounds on the overall hemostasis potential was determined using the CL-test through continuous measurement of changes in optical transmittance, as described previously [53,54]. The experiments were conducted using three-fold diluted human citrate plasma, t-PA and thrombin. The clot formation and lysis curves were determined at a wavelength of 405 nm using a CE 2021 spectrophotometer (Cecil, London, UK) with circulating thermostatic water (37 °C) and stirring. The results are presented as means ± SD (*n* = 3). The curves were used to determine the kinetic parameters of clot formation, stabilization and fibrinolysis using dedicated software [51]. The following parameters of clot formation were determined: Tt—thrombin time (s), Fmax—maximum clotting (%T), Tf—plasma clotting time (s), Fvo—initial plasma clotting velocity (%T/min), Sr—area under the clot formation curve (%Txmin). The parameters of the clot stabilization phase comprised Tc—clot stabilization time (s) and Sc—area under the curve of stable clot formation (%Txmin). The parameters of fibrinolysis comprised Lmax—maximum lysis (%T), Tl—fibrinolysis time (s), Lvo—initial clot fibrinolysis velocity (%T/min) and Sf—area under the fibrinolysis curve (%Txmin). Additionally, the overall potential for clot formation and fibrinolysis, i.e., CL_AUC_ (%Txmin), was also estimated.

#### 3.5.4. Erythrotoxicity

The influence of the compounds on the red blood cells (RBCs)’ membrane integrity was assessed using a lysis assay, as described previously [39]. Briefly, the tested compounds (1 to 100 μmol/L) were incubated in a 2% RBC suspension in 0.9% NaCl at 37 °C for 24 h; solvent was used as the control. The samples were then centrifuged at 3000× *g* for 10 min, and the absorbance of the supernatant was determined at 550 nm (maximum absorbance for hemoglobin). The results are presented as the degree of hemolysis, i.e., the percentage of released hemoglobin. A sample containing Triton X-100 was used as the positive control (100% hemolysis), whereas a sample of saline solution represented spontaneous hemolysis of the RBCs (control). The results are presented as means ± standard deviation (SD).

#### 3.5.5. Red Blood Cell Morphology

Various concentrations of the test compounds were incubated in 2% RBC suspension at 37 °C. The erythrocyte morphology was evaluated after one hour and 24 h using a phase contrast OPTA-TECH inverted microscope, at 400 times magnification, equipped for image analysis.

### 3.6. Statistical Analysis

The statistical analysis was performed using GraphPad Prism 5 software (San Diego, CA, USA). Statistically significant differences between the groups were identified using a one-way ANOVA test. The results were considered significant at *p*-values lower than 0.05.

## 4. Conclusions

Our study presents a possible correlation between the molecular structure and cytotoxic activity of Ag(I) complexes. Generally, the systems exhibiting a smaller HOMO-LUMO energy gap showed greater biological activity; these results also indicate that the metal–ligand bonds in these systems are characterized by a lower energy, i.e., they may be easier to break, and thus it may be easier for the silver ion to be released from the complex. The lengths of the Ag–N bonds in the crystalline state, as well as the observed activity of the nitrate(V) compounds, are consistent with the cytotoxicity evaluation.

The greatest selectivity index values, a particularly desirable feature for potential drugs, against the skin cancer and normal cells were observed for [Ag(MCZ)_2_NO_3_] (SI = 7.8) and [(MTZ)_2_AgNO_3_] (SI = 15.3). It is also noteworthy that the silver complex with MTZ was three-fold more toxic towards the A375 cells than the reference, cisplatin.

Our preliminary biocompatibility study revealed that [(MTZ)_2_AgNO_3_] and [(MTZ)_2_Ag]_2_SO_4_ do not cause pathological prolongation of the PT and INR beyond the reference range, even at high concentrations. Only metronidazole with Ag_2_SO_4_ at the highest concentration (100 µmol/L) showed a statistically significant prolongation of the APTT, and none of the tested compounds showed a significant impact on the TT. The compounds did not disrupt the clot formation process, its stabilization phase, nor lysis. The complexes of metronidazole with Ag(I) can be considered biocompatible (hemolysis below 10% after 24 h of incubation) at concentrations below 50 µmol/L.

Therefore, our preliminary studies provided a background for further research on the potential use of the studied complexes as pharmaceutical agents, which we plan to continue. Due to the limitations of [Ag(MCZ)_2_NO_3_] (has a pore solubility in water), research on [Ag(MTZ)_2_NO_3_] is more advanced. After all, [Ag(MTZ)_2_NO_3_] has been tested in clinics as part of medical experiments and is now used as a prescription drug prepared in pharmacies. Formulations of it as a drug (eye ointment and drops and ointment for external use) have been developed for the treatment of patients.

## Figures and Tables

**Table 1:** Results of crystallographic studies—geometrical parameters in the coordination sphere of the silver atom: d_1_, d_2_—Ag-N bond lengths [Å], α—N-Ag-N angle [°]; d—the distance of the Ag atom from the nearest ligand atoms.

Compound	d_1_	d_2_	α	d (Atom/Ligand)
[Ag(MCZ)_2_BF_4_]	2.058	2.058	176.47	3.225 (F/BF_4_^−^)
[Ag(MCZ)_2_ClO_4_]	2.068	2.068	175.81	3.140 (O/ClO_4_^−^)
[Ag(MCZ)_2_NO_3_]	2.181	2.181	152.39	2.511 (O/NO_3_^−^)
[Ag(MCZ)_2_SbF_6_]	2.092	2.092	180.00	3.303 (F/SbF_6_^−^)
[Ag(MTZ)_2_BF_4_]	2.110	2.112	174.86	3.885 (F/BF_4_^−^)
[Ag(MTZ)_2_CF_3_CO_2_]	2.177	2.160	162.73	2.480 (O/CF_3_CO_2_^−^)
[Ag(MTZ)_2_CH_3_SO_3_]	2.168	2.165	169.61	2.577 (O/ClO_4_^−^)
[Ag(MTZ)_2_ClO_4_]	2.126	2.128	175.81	2.660 (O/ClO_4_^−^)
[Ag(MTZ)_2_NO_3_]	2.147	2.147	165.30	2.639 (O/NO_3_^−^)
[Ag(MTZ) _2_]_2_SO_4_^#^	2.186	2.181	169.55	2.613 and 2.553 (O/hydroxyl group of MTZ) 2.928 and 2.954 (O/nitro group of MTZ)
[Ag(MTZ) _2_]_2_SO_4_^#^	2.197	2.197	170.50	2.634 and 2.591 (O/hydroxyl group of MTZ) 2.994 and 2.995 (O/nitro group of MTZ)

^#^ Crystal ([Ag (MTZ)]_2_SO_4_) contains two silver atoms in the symmetric unit.

**Table 2:** Ag-N bond lengths (d_1_ and d_2_, in Å) and N-Ag-N angle values (α, in °) for structures of complexes optimized at the ωB97X-D/def2-TZVPP level.

Compound	d_1_	d_2_	α
[Ag(MCZ)_2_BF_4_]	2.115	2.109	171.9
[Ag(MCZ)_2_ClO_4_]	2.112	2.116	175.3
[Ag(MCZ)_2_NO_3_]	2.176	2.176	164.8
[Ag(MCZ)_2_SbF_6_]	2.112	2.107	177.5
[Ag(MTZ)_2_BF_4_]	2.157	2.153	169.2
[Ag(MTZ)_2_CF_3_CO_2_]	2.205	2.198	157.3
[Ag(MTZ)_2_CH_3_SO_3_]	2.185	2.177	164.0
[Ag(MTZ)_2_ClO_4_]	2.142	2.146	179.1
[Ag(MTZ)_2_NO_3_]	2.196	2.196	160.6

**Table 3:** The values of electron density (ρ_1_ and ρ_2_, in e Å^−3^), Laplacian of electron density ($\nabla^{2}\rho_{1}$ and $\nabla^{2}\rho_{2}$, in e Å^−5^) and total energy density (hartree Å^−3^) for Ag-N bond critical points.

Compound	ρ_1_	ρ_2_	$\nabla^{2}\rho_{1}$	$\nabla^{2}\rho_{2}$	H_1_	H_2_
[Ag(MCZ)_2_BF_4_]	0.0909	0.0923	0.370	0.374	−0.0188	−0.0195
[Ag(MCZ)_2_ClO_4_]	0.0916	0.0906	0.372	0.369	−0.0191	−0.0187
[Ag(MCZ)_2_NO_3_]	0.0782	0.0782	0.325	0.325	−0.0130	−0.0130
[Ag(MCZ)_2_SbF_6_]	0.0914	0.0927	0.370	0.377	−0.0191	−0.0196
[Ag(MTZ)_2_BF_4_]	0.0820	0.0832	0.336	0.341	−0.0147	−0.0152
[Ag(MTZ)_2_CF_3_CO_2_]	0.0733	0.0744	0.304	0.310	−0.0112	−0.0115
[Ag(MTZ)_2_CH_3_SO_3_]	0.0767	0.0785	0.317	0.323	−0.0125	−0.0132
[Ag(MTZ)_2_ClO_4_]	0.0850	0.0841	0.350	0.337	−0.0160	−0.0158
[Ag(MTZ)_2_NO_3_]	0.0748	0.0748	0.310	0.310	−0.0117	−0.0117

**Table 4:** HOMO and LUMO energies and HOMO-LUMO energy gap (in eV).

Compound	E_HOMO_	E_LUMO_	ΔE_HOMO/LUMO_
[Ag(MCZ)_2_BF_4_]	−8.84	0.70	9.54
[Ag(MCZ)_2_ClO_4_]	−8.83	0.68	9.52
[Ag(MCZ)_2_NO_3_]	−7.40	0.39	7.78
[Ag(MCZ)_2_SbF_6_]	−8.53	0.40	8.93
[Ag(MTZ)_2_BF_4_]	−9.37	−1.04	8.32
[Ag(MTZ)_2_CF_3_CO_2_]	−8.75	−0.93	7.82
[Ag(MTZ)_2_CH_3_SO_3_]	−8.25	−0.91	7.34
[Ag(MTZ)_2_ClO_4_]	−9.00	−0.99	8.01
[Ag(MTZ)_2_NO_3_]	−7.77	−0.86	6.91

**Table 5:** Summary of the IC_50_ values of the tested compounds against A375 cancer cells and human fibroblasts BJ. Selectivity index (SI) was calculated using the formula SI = IC_50_ (BJ)/ IC_50_ (A375).

	IC_50_ (µM)		SI
	A375	BJ	
[(MTZ)_2_AgSO_4_]	22.8 ± 2.1	14.5 ± 0.8	0.63
[(MTZ)_2_AgNO_3_]	3.0 ± 0.3	46.0 ± 1.6	15.30
[Ag(MCZ)_2_NO_3_]	1.7 ± 0.1	13.3 ± 0.7	7.80
[Ag(MCZ)_2_ClO_4_]	19.8 ± 1.5	28.8 ± 2.1	1.45
[Ag(MCZ)_2_BF_4_]	20.4 ± 1.64	19.5 ± 1.2	0.96
[Ag(MCZ)_2_SbF_6_]	18.5 ± 2.4	16.0 ± 0.9	0.86
MCZ (miconazole)	12.8 ± 1.1	27.1 ± 0.8	2.12
MTZ (metronidazole)	>100	>100	-
Cisplatin	8.5 ± 0.3	24 ± 0.8	2.82
AgNO_3_	11.1 ± 0.8	>100	-

**Table 6:** The influence of [(MTZ)_2_AgNO_3_] on the overall potential for clot formation and fibrinolysis (CL_AUC_) and its parameters; data are presented as means and standard deviation (SD; *n* = 3). The compound did not significant affect the overall potential for clot formation and fibrinolysis (CL_AUC_) or any of the evaluated kinetic parameters over the entire concentration range.

[(MTZ)_2_AgNO_3_]		Tt [s]	Fmax [%]	Tf [s]	Fvo [%/min]	Tc [s]	Lmax [%]	Tl [s]	Lvo [%/min]	Sr [%*min]	Sc [%*min]	Sf [%*min]	CL_AUC_ [%*min]
CTR	mean	18.9	63.4	71.2	113.7	123.7	62.3	180.0	190.0	48.9	126.4	114.6	289.9
	SD	4.47	2.10	4.80	19.62	4.16	2.21	5.29	1.30	3.68	3.05	7.56	7.02
0.5 µmol/L	mean	21.3	64.4	61.9	126.7	114.3	61.9	158.7	20.9	44.1	118.4	125.5	287.9
	SD	5.84	1.21	7.43	14.57	4.04	3.86	6.81	1.96	4.90	4.82	39.34	41.06
1.0 µmol/L	mean	21.6	62.2	65.9	126.3	124.7	61.5	175.0	19.5	45.1	125.5	105.1	275.7
	SD	4.33	7.80	10.64	37.45	9.81	8.58	11.27	3.67	1.16	22.73	12.44	28.98
5.0 µmol/L	mean	19.2	70.7	59.4	157.7	146.7	70.5	199.0	21.9	44.7	169.8	129.1	343.6
	SD	7.41	7.51	9.33	56.50	28.75	7.48	24.06	1.51	1.41	51.72	31.40	83.69
10.0 µmol/L	mean	23.3	61.4	62.3	121.6	126.7	60.2	180.3	19.5	40.5	126.7	117.2	284.4
	SD	3.17	6.44	11.87	40.67	17.79	9.15	26.10	4.37	5.43	29.04	29.71	34.06
50.0 µmol/L	mean	22.5	63.8	56.9	129.0	119.3	61.8	164.7	22.0	38.9	123.25	118.6	280.7
	SD	5.42	7.42	7.68	15.87	6.11	6.87	15.95	3.29	3.75	20.73	28.33	46.92
100.0 µmol/L	mean	22.4	68.6	59.2	124.9	131.3	67.2	188.7	29.6	40.4	146.3	131.8	318.6
	SD	4.18	6.38	3.56	31.38	13.61	4.78	18.90	13.46	4.83	27.01	52.16	78.60

CTR—control sample, Tt—thrombin time (s), Fmax—maximum clotting (%T), Tf—plasma clotting time (s), Fvo—initial plasma clotting velocity (%T/min), Sr—area under the clot formation curve (%Txmin), Tc—clot stabilization time (s), Sc—area under the curve of stable clot formation (%Txmin), Lmax—maximum lysis (%T), Tl—fibrinolysis time (s), Lvo—initial clot fibrinolysis velocity (%T/min), Sf—area under the fibrinolysis curve (%Txmin), overall potential for clot formation and fibrinolysis (CL_AUC_ (%Txmin)).

**Table 7:** Influence of [(MTZ)_2_Ag]_2_SO_4_ on overall potential for clot formation and fibrinolysis (CL_AUC_) and its associated parameters, presented as means and standard deviation (SD; *n* = 3). The compound did not significantly affect the overall potential for clot formation and fibrinolysis (CL_AUC_) or any of the evaluated kinetic parameters over the entire concentration range.

[(MTZ)_2_Ag]_2_SO_4_		Tt [s]	Fmax [%]	Tf [s]	Fvo [%/min]	Tc [s]	Lmax [%]	Tl [s]	Lvo [%/min]	Sr [%*min]	Sc [%*min]	Sf [%*min]	CL_AUC_ [%*min]
CTR	mean	18.2	78.4	65.4	191.3	176.7	77.7	224.3	21.9	59.7	223.5	169.4	452.5
	SD	4.24	2.71	8.01	25.58	7.64	2.18	12.58	0.56	9.31	10.73	15.24	23.35
0.5 µmol/L	mean	20.1	70.6	59.1	160.0	144.3	69.8	196.7	20.3	47.3	165.1	135.9	348.3
	SD	2.77	6.79	6.23	21.93	10.97	6.82	12.06	1.79	6.56	27.21	20.20	50.36
1.0 µmol/L	mean	20.5	73.8	61.2	181.0	174.3	73.5	226.3	19.8	52.4	207.8	157.6	417.7
	SD	1.93	2.74	1.16	24.52	5.69	2.80	12.66	0.42	3.17	9.83	4.41	15.01
5.0 µmol/L	mean	19.7	78.6	50.2	212.3	166.7	77.9	204.7	24.8	44.8	210.7	155.5	411.1
	SD	2.15	4.50	9.40	45.52	24.01	4.30	41.49	5.47	5.94	21.16	20.69	46.24
10.0 µmol/L	mean	20.7	75.7	55.0	178.3	166.7	75.0	208.3	21.5	46.4	203.7	156.2	406.3
	SD	2.46	2.28	3.03	9.81	14.84	3.30	22.19	0.55	1.42	20.73	11.99	24.52
50.0 µmol/L	mean	20.7	76.9	58.4	159.6	158.7	74.3	200.7	21.7	47.5	184.0	175.2	421.1
	SD	7.07	7.64	4.03	21.73	17.62	5.98	18.45	1.50	2.47	42.57	61.69	93.01
100.0 µmol/L	mean	20.5	72.3	62.6	159.3	166.3	72.0	219.7	22.1	50.8	194.4	145.2	390.3
	SD	5.25	3.33	6.95	17.93	16.56	1.18	3.79	4.97	7.36	24.30	33.95	62.11

CTR—control sample, Tt—thrombin time (s), Fmax—maximum clotting (%T), Tf—plasma clotting time (s), Fvo—initial plasma clotting velocity (%T/min), Sr—area under the clot formation curve (%Txmin), Tc—clot stabilization time (s), Sc—area under the curve of stable clot formation (%Txmin), Lmax—maximum lysis (%T), Tl—fibrinolysis time (s), Lvo—initial clot fibrinolysis velocity (%T/min), Sf—area under the fibrinolysis curve (%Txmin), overall potential for clot formation and fibrinolysis (CL_AUC_ (%Txmin)).

## Data Availability

The data presented in this study are available on request from the corresponding author.

## Supplementary Materials

The following supporting information can be downloaded at https://www.mdpi.com/article/10.3390/ijms25105081/s1.
