# Supplementary material for: Silver Complexes of Miconazole and Metronidazole: Potential Candidates for Melanoma Treatment
Source: Int J Mol Sci. 2024 May 7;25(10):5081. doi: 10.3390/ijms25105081 (PMC11121064; doi:10.3390/ijms25105081)
Supplement: Supplementary file 1 [file ijms-25-05081-s001.zip › ijms-2981473-supplementary.pdf]

Silver Complexes of Miconazole and Metronidazole: Potential Candidates for Melanoma Treatment

Małgorzata Fabijańska [1,*], Agnieszka J. Rybarczyk-Pirek [2], Justyna Dominikowska [2], Karolina Stryjska[1], Dominik Żyro[1], Magdalena Markowicz-Piasecka [3], Małgorzata Iwona Szynkowska-Jóźwik [4], Justyn Ochocki [5] and Joanna Sikora[1]

[1] Department of Bioinorganic Chemistry, Medical University of Lodz, ul. Muszynskiego 1, 90-151 Lodz,
Poland
[2] Theoretical and Structural Chemistry Group, Department of Physical Chemistry, Faculty of Chemistry, University of Lodz, Pomorska 163/165, 90-236 Łódź, Poland
[3] Department of Applied Pharmacy, Medical University of Lodz, ul. Muszynskiego 1, 90-151 Lodz, Poland
[4] Faculty of Chemistry, Institute of General and Ecological Chemistry, Lodz University of Technology, Zeromskiego 116, 90-543 Lodz, Poland
[5] Faculty of Pharmacy, Chair of Medicinal Chemistry, Group of Bioinorganic Chemistry Medical University of Lodz, 1 Muszynskiego Str., 90-151 Lodz, Poland (Professor Emeritus of Pharmacy)
* Correspondence: e-mail: malgorzata.fabijanska@umed.lodz.pl; Tel.: +48426779222

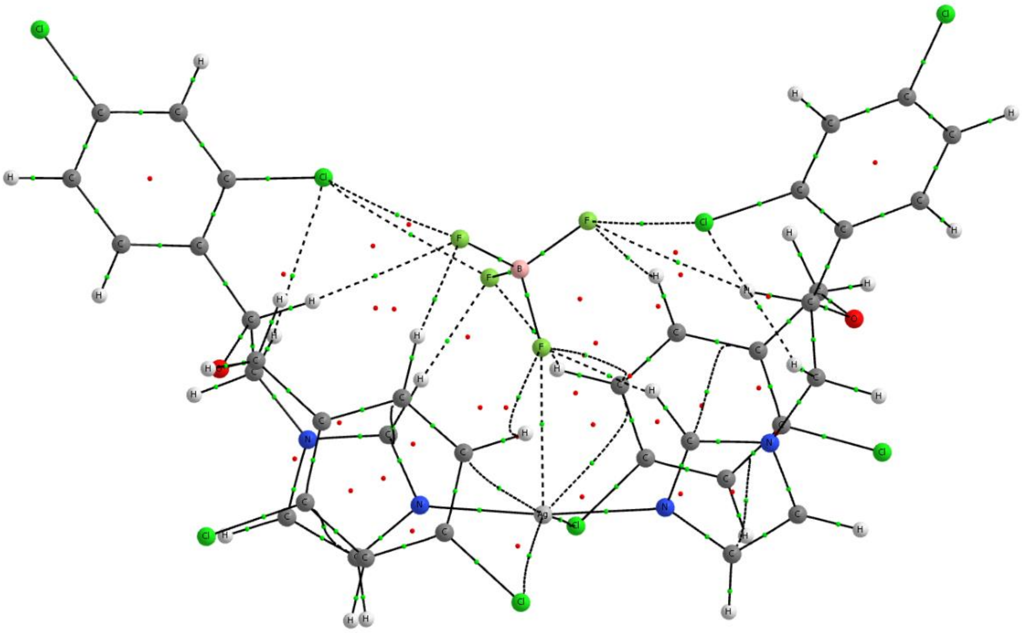

Figure S1. Molecular graph of $Ag(MCZ)_2BF_4$; green dots – bond critical points, red dots – ring critical points, cage critical points are omitted for picture clarity.

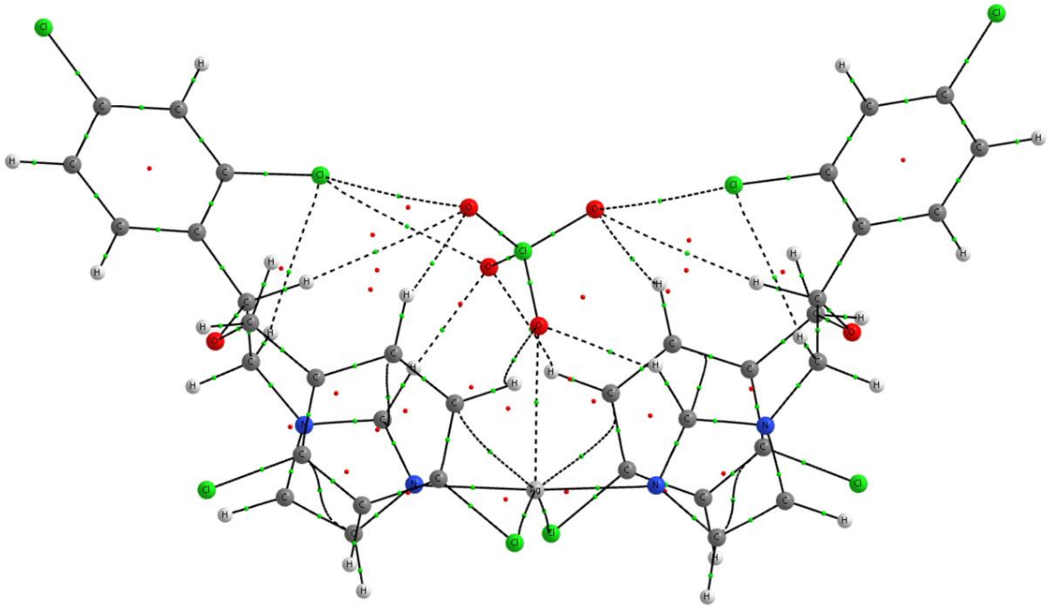

Figure S2. Molecular graph of $Ag(MCZ)_2ClO_4$; green dots – bond critical points, red dots – ring critical points, cage critical points are omitted for picture clarity.

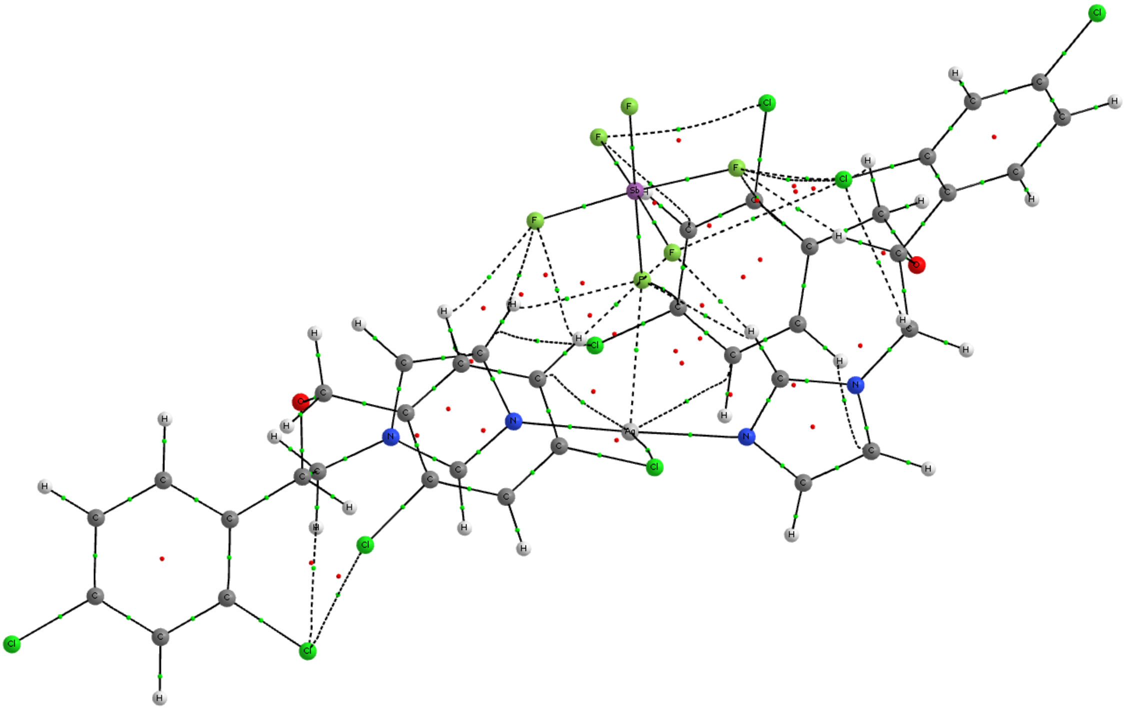

Figure S3. Molecular graph of $Ag(MCZ)_2SbF_6$; green dots – bond critical points, red dots – ring critical points, cage critical points are omitted for picture clarity.

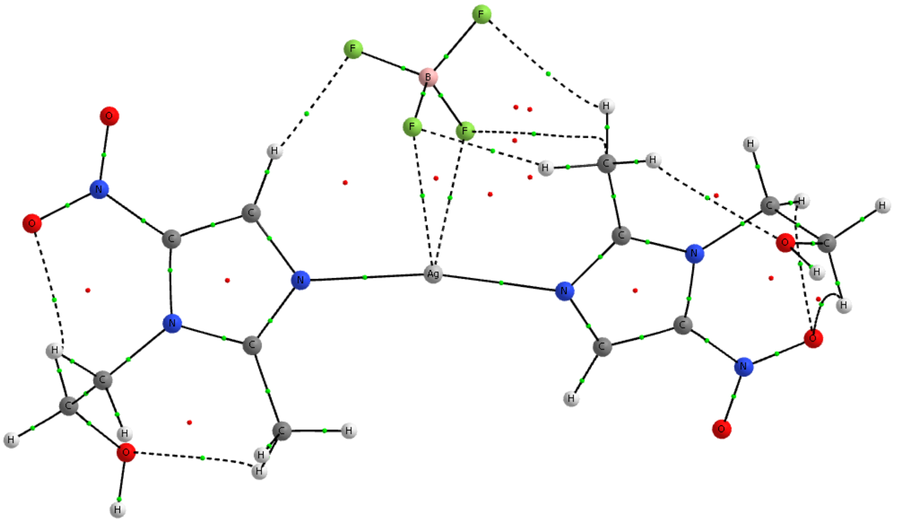

Figure S4. Molecular graph of $Ag(MTZ)_2BF_4$; green dots – bond critical points, red dots – ring critical points, cage critical points are omitted for picture clarity.

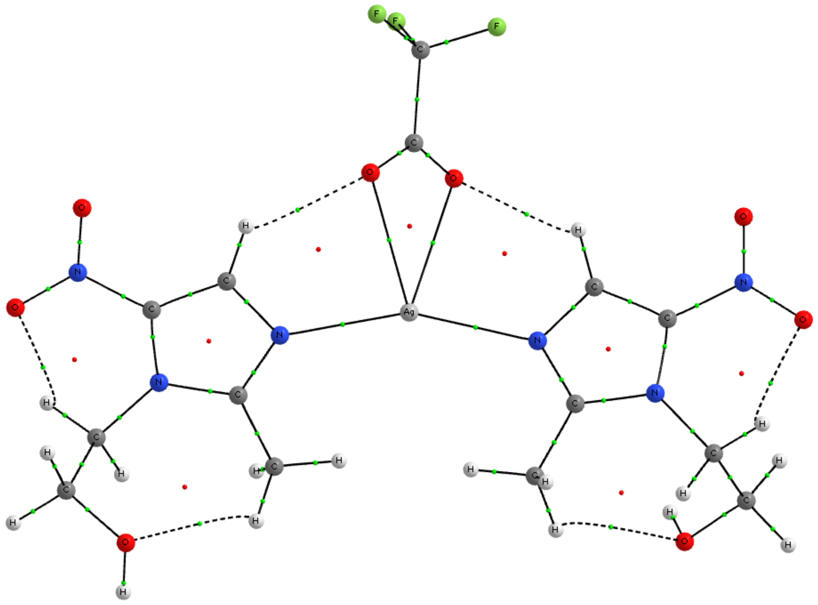

Figure S5. Molecular graph of $Ag(MTZ)_2CF_3CO_2$; green dots – bond critical points, red dots – ring critical points, cage critical points are omitted for picture clarity.

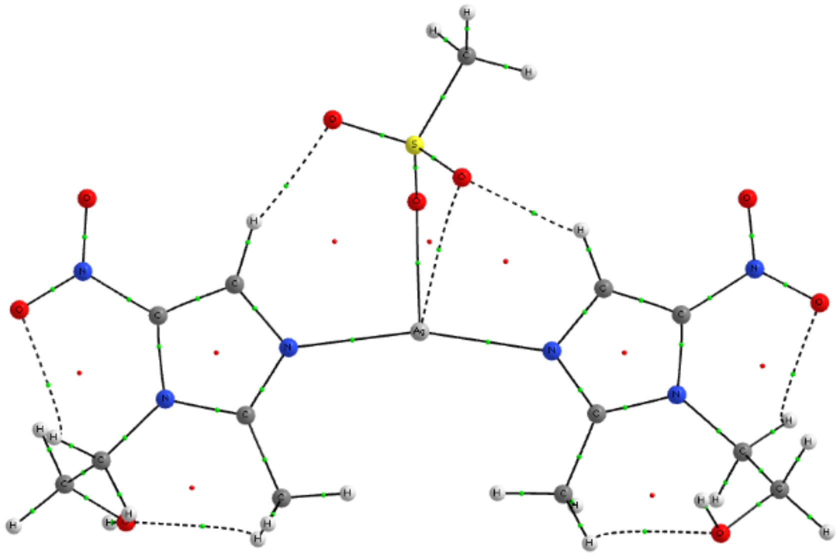

Figure S6. Molecular graph of Ag(MTZ)$_2$CH$_3$SO$_3$; green dots – bond critical points, red dots – ring critical points, cage critical points are omitted for picture clarity.

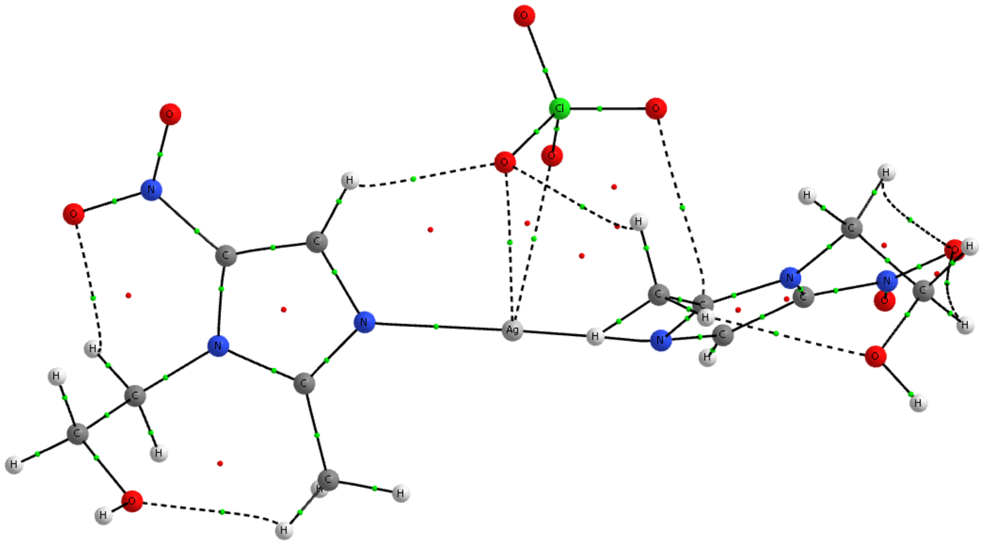

Figure S7. Molecular graph of Ag(MTZ)$_2$ClO$_4$; green dots – bond critical points, red dots – ring critical points, cage critical points are omitted for picture clarity.

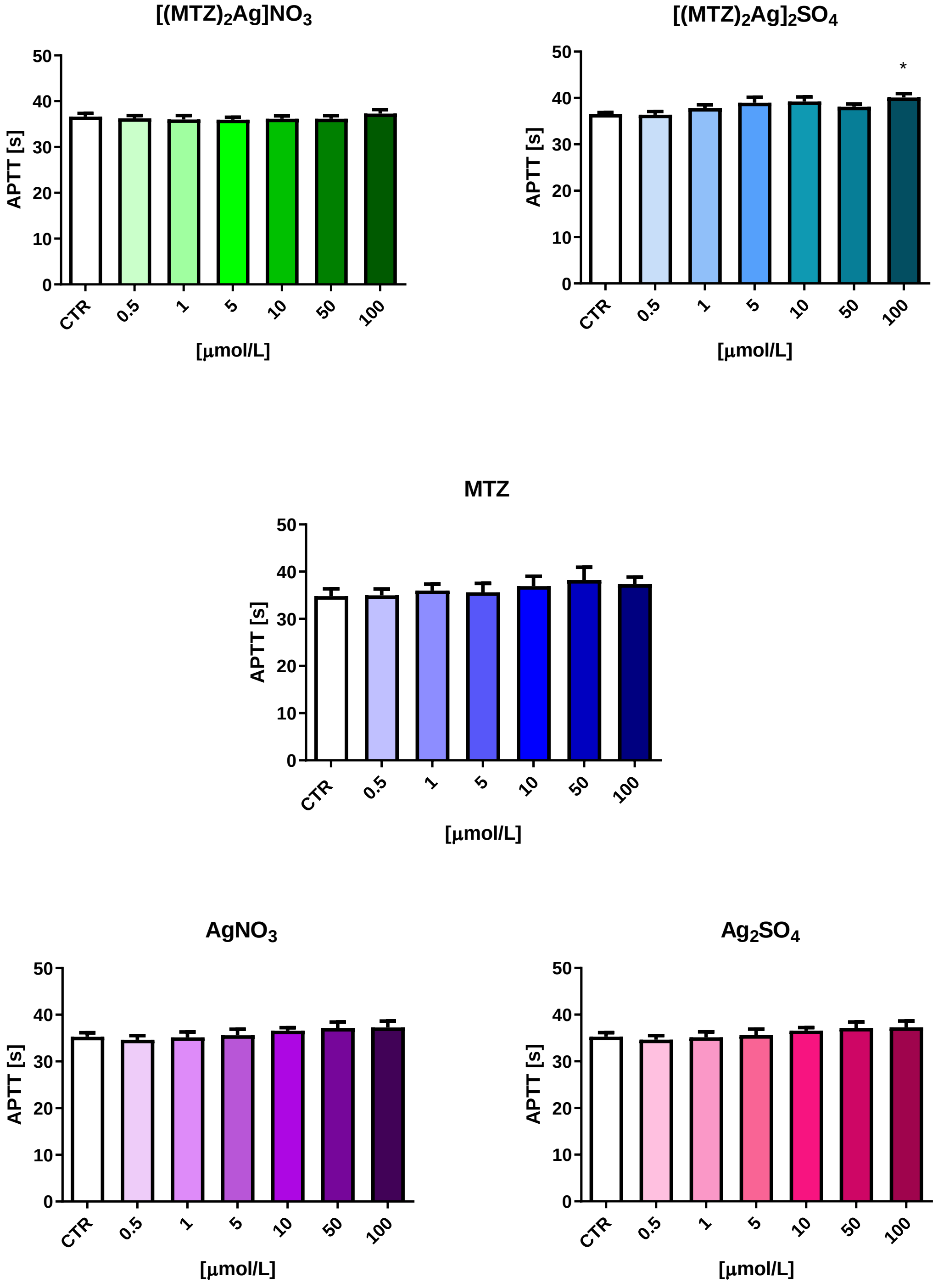

Figure S8. The influence of the tested silver(I) metronidazole complexes ([(MTZ)2Ag]NO3 and [(MTZ)2AgSO4]) and ligands (MTZ - metronidazole; $AgNO_3$; Ag_2SO_4) on activated partial thromboplastin time (reference range: 26.7-40.0 s). The results are presented as mean ± standard deviation (n = 5); * $p < 0.05$ there is a statistically significant difference compared to the control sample (CTR).

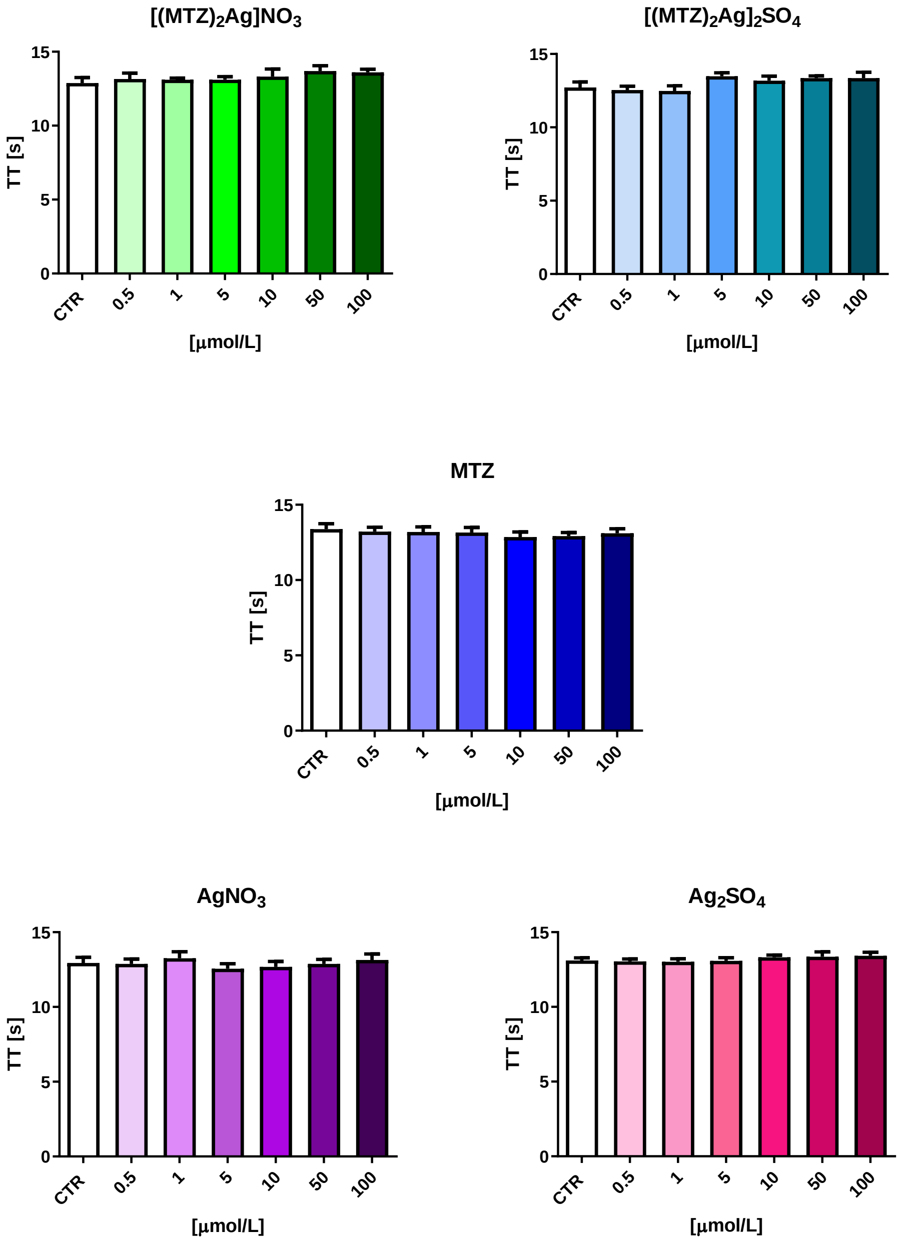

Figure S9. The influence of the tested silver(I) metronidazole complexes ([(MTZ)2Ag]NO3 and [(MTZ)2AgSO4]) and ligands (MTZ - metronidazole; $AgNO_3$; Ag_2SO_4) on thrombin time". (reference range: 14.0-18.0 s). The results are presented as mean ± standard deviation (n = 5); control sample (CTR).

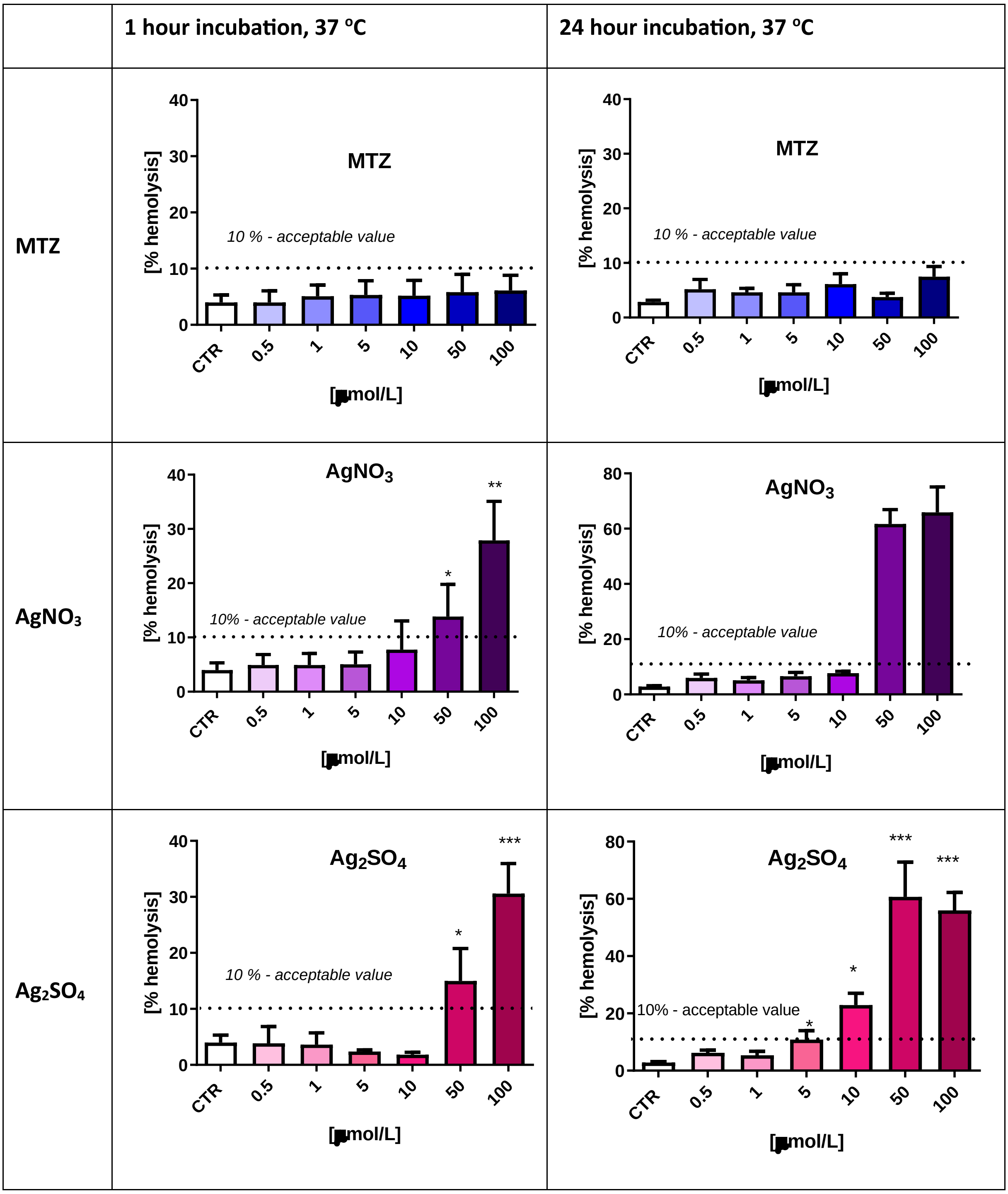

Figure S10. The effects of incubation 2 % RBC suspension with ligands (MTZ - metronidazole; $AgNO_3$; Ag_2SO_4) on RBC hemolysis (acceptable value of hemolysis proving the biocompatibility of the compound is 10%). The results are presented as mean ± standard deviation (n = 35); * $p < 0.05$, ** $p < 0.01$, *** $p < 0.01$ there is a statistically significant difference compared to the control sample (CTR).
